# Nutrigenomics of Body Weight Regulation: A Rationale for Careful Dissection of Individual Contributors

**DOI:** 10.3390/nu6104531

**Published:** 2014-10-21

**Authors:** Jaap Keijer, Femke P. M. Hoevenaars, Arie Nieuwenhuizen, Evert M. van Schothorst

**Affiliations:** Human and Animal Physiology, Wageningen University, De Elst 1, 6708 WD Wageningen, The Netherlands; E-Mails: f.hoevenaars@vumc.nl (F.P.M.H.); arie.nieuwenhuizen@wur.nl (A.N.); evert.vanschothorst@wur.nl (E.M.S.)

**Keywords:** overweight, obesity, body weight regain, body weight loss, setpoint, settling point, study design, leptin

## Abstract

Body weight stability may imply active regulation towards a certain physiological condition, a body weight setpoint. This interpretation is ill at odds with the world-wide increase in overweight and obesity. Until now, a body weight setpoint has remained elusive and the setpoint theory did not provide practical clues for body weight reduction interventions. For this an alternative theoretical model is necessary, which is available as the settling point model. The settling point model postulates that there is little active regulation towards a predefined body weight, but that body weight settles based on the resultant of a number of contributors, represented by the individual’s genetic predisposition, in interaction with environmental and socioeconomic factors, such as diet and lifestyle. This review refines the settling point model and argues that by taking body weight regulation from a settling point perspective, the road will be opened to careful dissection of the various contributors to establishment of body weight and its regulation. This is both necessary and useful. Nutrigenomic technologies may help to delineate contributors to body weight settling. Understanding how and to which extent the different contributors influence body weight will allow the design of weight loss and weight maintenance interventions, which hopefully are more successful than those that are currently available.

## 1. Introduction: Obesity and Body Weight Loss

The number of people who are obese is still rising globally. In 2012 in the Netherlands, approximately 53% of the male population and 44% of the female population, above the age of 20, was overweight (body mass index (BMI) > 25 kg/m^2^) and 11% and 14%, respectively, were obese (BMI > 30 kg/m^2^) [1]. These Dutch figures are not an exception, in fact the number of people who are overweight or obese are higher in many countries and are increasing world-wide in many others, often at alarming rates [2,3]. With the increased prevalence of overweight and obese people, also the number of people attempting to lose body weight increases. Several body weight loss strategies for humans exist, some more successful than others. Diets with reduced energy content or weight loss medication are generally successful in reaching a body weight loss of 5%–9% with body weight plateauing after 6 months [4]. Also, reduction of a specific macronutrient, such as fats (e.g., [5]) or carbohydrates (e.g., [6]), is considered as an effective tool for body weight loss [7,8]. Body weight reduction is considered significant when 5%–10% of the initial body weight is lost, as this is associated with an improvement of metabolic and cardiovascular health parameters [9,10]. A 5%–10% body weight reduction is realized in many instances within 3–18 months [11]. Unfortunately, in most cases the main problem with dieting is not the achievement of short term weight loss, but is the long term body weight maintenance after body weight reduction. Indeed, after 2.5 years of follow-up, participants from four different intervention groups aiming to lose body weight on average only lost 1.7 kg (1.4−2.2 kg, depending on the intervention) compared to their body weight at the start of the intervention [12], while weight loss after 6 months varied between 8 and 11.2 kg in the different intervention groups indicating that the long term success rate of body weight reduction is substantially lower than the initial success. This is supported by a meta-analysis of body weight reduction studies, in which it was observed that after 48 months half of the body weight reduction had disappeared [4]. Also, the National Health and Nutrition Examination Survey (NHANES) study shows that dieters are not in the advantage in the long run. Thirty-five percent of people with significant reduced body weight regained body weight after one year [13]. The regain of body weight is thought to reflect regulation towards a fixed body weight setpoint. As mentioned, the biggest problem with body weight reduction is maintenance of a lower body weight; while short term effectiveness of dieting is frequently high, long term maintenance seems more difficult. Successful maintenance of body weight loss is dependent on several factors; it starts with continued dietary restraint, but it also includes frequent self-monitoring, undertaking regular exercise, and limiting inactive behaviors, such as watching television [14].

## 2. Energy Balance

**Figure 1:**
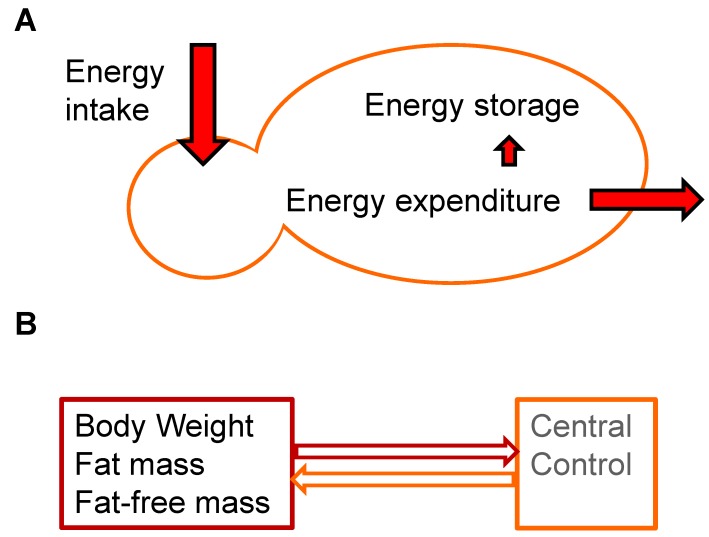
Body energy balance and its regulation according to the setpoint model. (**a**) Balance between energy intake and energy expenditure. Excess energy is stored primarily in white adipose tissue causing overweight and obesity; (**b**) The setpoint model for body weight regulation represents a closed loop model in which the controlled quantity can be body weight, fat mass, or fat free mass (**left**) which sends a signal to the central control system (**right**). The central control system in turn sets body weight and body composition.

Body energy balance is often represented by a simple formula: energy intake = energy expenditure + energy storage, with energy storage being either neutral, positive (body weight gain) or negative (body weight loss) (Figure 1A). This is essentially a restatement of the first law of thermodynamics, since energy cannot be created nor destroyed. When we disentangle this energy balance formula, it is evident that food is the main source of our energy intake. Energy expenditure can be divided into three main categories, being resting metabolic rate (RMR, *i.e.*, energy needed for maintenance of physiological functions), adaptive thermogenesis (*i.e.*, energy expended for adaptive processes such as digestion, uptake and processing of ingested macro nutrients or energy expended to adjust to environmental temperature), and physical activity (*i.e.*, energy used for conscious facultative activities such as the use of muscles for exercise) [15,16]. Surplus energy is stored as triglycerides, primarily in white adipose tissue (WAT). WAT is a metabolically and endocrine active tissue that is well equipped for energy storage [17]. It protects other tissues from fatty acid overload. Long term chronic energy excess is associated with adipose tissue dysfunction, contributing to obesity associated metabolic diseases [18]. Remarkably, despite substantial daily variation in energy intake and expenditure, most adults obtain a relatively stable body weight [2,19]. Individual body weight variance is around 0.5% over periods of 6–10 weeks [20]. Net energy balance is counteracted by changes in food intake and/or energy expenditure to minimize changes in body weight [21]. Weight stability suggests homeostatic control, but homeostasis implies active regulation towards a certain physiological condition. From the late 1970s–early 1980s the percentage of overweight people started to rise and it seems that we are not able to “auto regulate” our body weight around a fixed point any longer. Furthermore, obesity prevalence varies for different societal strata (e.g., [22,23,24]) and is associated with consumption of various processed foods (e.g., [25,26]). This casts doubts on the fixed setpoint theory, as the setpoint seems to move upward and susceptibility is dependent on socio-economic status and other societal factors.

## 3. Contributors to Body Weight Gain

One way to explain the obesity epidemic is by the thrifty gene hypothesis. It starts from the assumption that through natural selection we evolved to be efficient in energy storage for times of famine [27]. Next to this hypothesis, overabundance of food (e.g., introduction of fast food and soft drinks), a sedentary lifestyle (e.g., transportation systems), labor saving technologies (e.g., remote controls, machines and robots) and increased environmental protection and buffering (e.g., improved housing and temperature controlled working environments) are factors that make us susceptible to development of obesity and its associated pathologies. Interaction of these factors results in a dynamic equilibrium, a “settling point”, which is dependent on the constitution of the individual [28], in interaction with current energy input and output [29]. Individuals that develop obesity in childhood due to dominant genetic mutations (e.g., [30]) can be considered an extreme case, where their constitution overrides environmental factors. However, this group of people represents a very small subset of all obese and will not further be considered here.

## 4. Setpoint

The setpoint model can be considered as a cruise control; when the system is disrupted by either losing or gaining weight, the weight will be either regained or lost to the original settings. Body weight regulation might be secondary to regulation of a component of our body composition, such as total fat. This led to the “lipostatic” theory in which total body fat regulation determines body weight [31]. As mentioned before, the setpoint model represents a closed loop model in which the controlled quantity can be fat mass, body weight or even fat free mass which sends a signal to the central control system (Figure 1B). The value of the setpoint is independent of the operation of the system, and, once set, remains the same until readjusted [29]. It is now clear that this might be an oversimplified model as more factors become known which are able to influence body weight and body composition.

## 5. Settling Point

**Figure 2:**
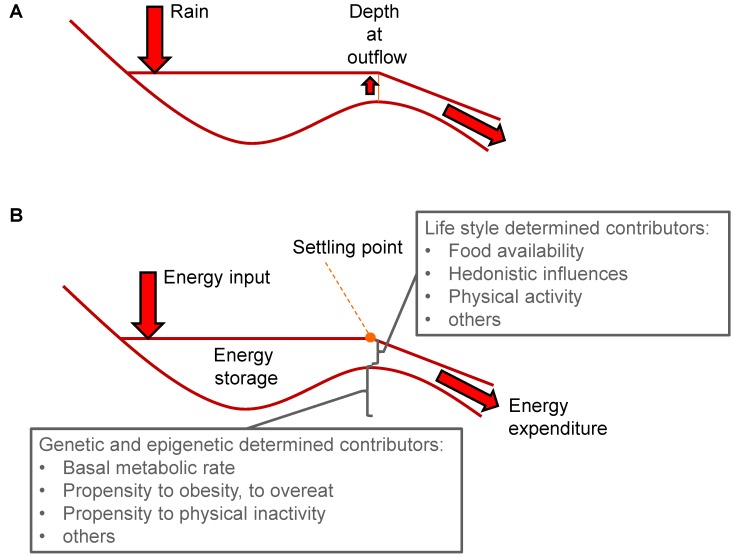
Body weight regulation according to the settling point model. (**a**) The settling point model is based on a natural equilibrium. As an example, a reservoir, here a lake, will show an adjustable total volume of water. Due to extra inflow of water, the water level rises until water outflow equals water inflow. In addition to the amount of rain the volume of the lake is determined by size of the lake and size of outflow. Changes can be buffered by, for example, overflow areas. This panel is adapted from [32]; (**b**) The scheme of panel 2a translated into regulation of body weight. Rain is translated into energy input. The lake represents body energy stores and outflow represents energy expenditure. Depth at outflow represents the settling point. Overflow areas represent resistance to weight loss. The individual’s constitution, determined by genetic and epigenetic contributors, determines the propensity to become overweight, while life style contributors, such as food availability, energy density and palatability as well as physical activity, determine variability in energy storage. Together these determine the settling point value.

In the settling point model, it is assumed that there is little active predefined regulation of body weight, but that this settles determined by environmental and socioeconomic factors, such as diet and lifestyle, in interaction with genetic pre-disposition, or, to phrase this more generally, in interaction with the individual’s constitution. Precise regulation takes place without a fixed setpoint, rather body weight settles based on that resultant of a number of contributors. An analogy for body weight regulation in the settling point model is the level of water in a lake [32]. A natural equilibrium is present in a reservoir, here a lake, due to extra inflow of rain, water rises until outflow equals inflow (Figure 2A). When translated into regulation of body weight, body energy stores represent the lake while rain is translated into energy input, and depth at outflow represents energy expenditure (Figure 2B). The lake equals energy storage.

## 6. The Rise in Obesity Questions a Body Weight Setpoint

As described above, a setpoint is a target value of a controlled variable that is maintained by an automatic control system. This is analogous to a cruise control system in which a certain target speed is programmed; when driving up a mountain, the internal control system of the car will hit the gas to retain the target speed, but when driving down the car will hit the brakes to return to its target speed. One big difference between a physiological setpoint and the cruise control system is that you can tune the cruise control to your own desire, but a physiological setpoint is fixed. Such a fixed setpoint contrasts with the rise in obesity; did we collectively release such a fixed setpoint and did we move our “fixed” setpoint upwards?

## 7. Setpoint *versus* Settling Point

**Figure 3:**
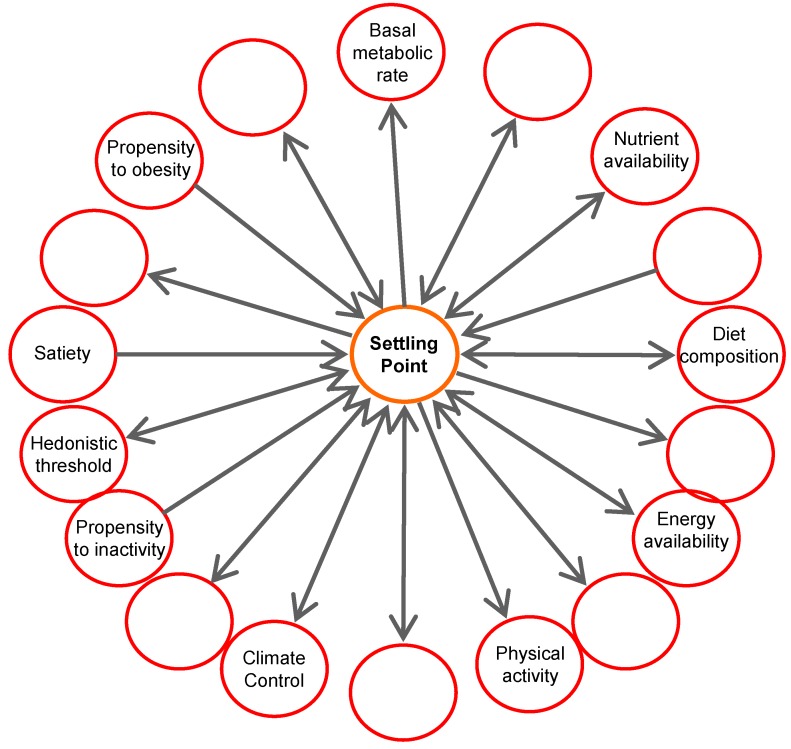
Contributors to body weight settling. The position of the settling point represents optimal functioning for a certain set of contributors, which can be considered as forces in a sphere. Different forces are able to dynamically influence the settling point, such as energy and nutrient availability, metabolic rate, and maintenance of the organism in terms of metabolic flexibility and hedonism. Established contributors may be used as functional targets for nutrients.

It is virtually impossible to assess setpoint and settling point theories in humans, mainly because it is not possible to change only one variable at a time. Using highly controlled mouse studies, we and others have shown that changes from a high energy to low energy diet and the reverse, did not result in a persistent elevated body weight setpoint when purified diets with identical ingredients were used [33,34]. The change from one diet to another resulted in partial compensation by adjustment of feed intake. However, this was not sufficient for weight maintenance. These studies rather show that the last consumed diet, either high or low in fat content, determine energy intake, energy expenditure, body weight, body fat mass, and circulating hormones and metabolites [34]. Similar results have been obtained in humans: subjects that switched from a normal fat diet to a low fat diet eaten *ad libitum* lost body weight, but regained their body weights once they returned to normal fat diets [35]. This agrees with studies in twins that did not show a persistent effect of overfeeding [36]. Based on these results, we concluded that all these parameters “settle” into a new flexible point determined by energetic input and output, thus rejecting the setpoint theory and supporting the settling point theory. Nevertheless, also in the settling point theory the question remains: which factors are responsible for establishment of the settling point? In other words, which factors are responsible for keeping weight balance? It is unlikely that this is a single contributor. Most likely, it is the optimal balance, within a certain sphere, between a number of genetically determined parameters [37], delineating the individual’s constitution, driven by environmental and life style parameters, demarcating current energy input and output (Figure 2B). Parameters that delineate the individual’s constitution include those that determine metabolic rate, body size, body composition, metabolic efficiency and propensity to physical activity, while energy and nutrient availability, palatability of food, physical activity and living and working environment can be considered as modifying factors. These different contributors interact and together determine the point where body weight settles. This is schematically visualized in Figure 3. Clearly as part of these contributors feedback mechanisms operate. These mechanisms may respond to a variety of cues, resulting in modified behavior and physiology, affecting body weight, but not necessarily stemming from the need to defend body weight. An important practical aspect of identification of individual contributors is that bona fide contributors can be used as a functional parameter to screen beneficial effects of nutrients, functional foods or ingredients.

## 8. Forward, Taking the Settling Point Perspective

From feeding experiments in mice we have learned that when we feed the animals with diets composed of identical ingredients, but differing in the amount of fat relative to carbohydrates, that diet itself has a major influence on weight balance [33,34]; a diet specific response, *i.e.*, a high fat diet or low fat diet determined response, was observed for all measured parameters. These studies differ in outcome from studies where a purified high fat diet was compared to a low fat chow diet, which showed programming effects of the high fat diet, suggesting a body weight setpoint [38]. Viewed from another perspective, this rather suggests that various components of the diets, such as protein composition, modify the inclination to become obese. This is supported by the concept of non-equilibrium thermodynamics, and a wealth of underlying data, essentially indicating that not only the amount of calories, but also diet dependent differences in metabolic flux may result in net body weight gain or body weight loss [39,40]. Metabolic fluxes can occur at the whole body level, but can also play a role at the tissue level. In WAT, for example, continuous intracellular lipid cycling occurs, consisting of breakdown of tri-acylglycerides into fatty acids and glycerol and re-esterification of these fatty acids, as acyl-CoA, with glycerol-3-phosphate back into tri-acylglycerides. This process dissipates energy as it requires ATP. Although glycerol-3-phosphate in WAT is primarily generated via glyceroneogenesis [41], it has long been established that glycerol kinase is also active in WAT [42] and its activation has been suggested as one of the mechanisms underlying the beneficial action of the thiazolidinediones (TZD) class of anti-diabetic drugs [43]. Regulation of substrate fluxes are fully dependent on dietary composition and may additionally be affected by specific dietary constituents, such as long-chain omega-3 poly-unsaturated fatty acids, which exert their effect via the pivotal AMP protein kinase (AMPK) cellular energy sensor (reviewed in [44]), which regulates many of these processes [45]. The physiological importance of these fluxes for body weight regulation is emphasized by the observation that obesity-resistant rats show higher levels of dietary fat oxidation and lower levels of dietary fat storage than obesity-prone rats [46], which may be explained by the satiating effects of hepatic fat oxidation [47].

The setpoint interpretation of body weight regulation directs further research towards identification of such a setpoint, a quest that until now has largely been unproductive and ill at odds with the societal increase in obesity, differing for societal strata and being dependent on society type (e.g., the effect of migration to a more affluent society [48]). On the other hand, when bodyweight regulation studies are interpreted from a settling point perspective, studies different in weight regain are not conflicting, but rather suggest that it is both necessary and useful to carefully dissect various contributors to establishment of body weight and its regulation. Understanding the manner and extent to which the different contributors influence body weight will then allow the design of weight loss and weight maintenance interventions which are more successful than those we have currently available.

## 9. Study Design; Detailes Matter

Studies to dissect different contributors to body weight regulation are not easy to design or perform. Taking one of our own mouse studies, where we examined body weight loss by two standard strategies, will exemplify this. In this study, we compared a reduction of portion size (30% energy restriction of a high fat diet) with a restriction of dietary fat intake (change from *ad libitum* high fat diet to *ad libitum* low fat diet) [49]. These interventions were performed with purified diets containing the same ingredients, with only the amounts (but not the type) of carbohydrates and fats differing between the high fat and low fat diets. Both strategies reduced energy intake. We observed improved health parameters, including lower adipose tissue inflammation, in the restricted group compared to the low fat diet group [49]. However, our choice for comparing two widely used interventions, rather than focusing on equal (reduced) energy intake, led to a different energy intake between the two weight loss groups, in addition to the inherently different macronutrient intake (fat to carbohydrate ratio). Moreover, because the low fat diet was being fed *ad libitum* while the high fat diet was fed in a restricted manner prior to the start of the dark phase, this may have resulted in altered physical activity behavior, since restricted mice tend to be more physically active just prior to feeding. With hindsight it is easy to see that addition of a pair-fed control group (fed the same amount of calories of the low fat diet as the high fat restricted diet) would have resulted in an equal amount of energy intake and would have negated possible differential effects of restriction on physical activity. It is less easy to accommodate differences in macronutrient ratios. This was attempted in a study performed with female mice where reduction of dietary energy density reduced body weight regain after energy restriction [50]. In this study, the energy density of the food was reduced by the addition of cellulose, which thus resulted in a reduction of net energy intake, but it also resulted in a probable reduction in macro- and micronutrient intake and it may have affected microbiota as well as food transition time. This example further underlines the difficulty to separate contributors to a body weight settling point. Nevertheless, we should try, preferably using common standardized reference diets [51,52].

Careful assessment of the influence of various dietary factors, such a nutrient composition and palatability, on body weight, body weight loss and body weight regain is difficult in mice and rats. Likely it is even more difficult in humans, because, for example, diet manipulation and adherence monitoring are more complicated. This, however, is no argument against trying, especially since delineation of the role and effect of different contributors is essential in the design of effective weight loss and weight maintenance interventions.

## 10. Weight Loss, Metabolic Rate and Leptin

It has been observed that weight loss in mice [53,54] and in humans [21,55,56,57,58,59,60] is accompanied by reductions in metabolic rate (per kg of fat free mass) below the level that would be expected based on their lower body weight as a result of energy restriction. This drop in metabolic rate promotes the conservation of body energy by sparing lean and fat mass. This may also predispose to a positive weight balance. The body weight loss mediated reductions in metabolic rate were shown to extend well beyond the dynamic period of body weight loss [61,62] and have even been observed to remain present in humans for up to six years [63]. A persistent reduction in metabolic rate due to body weight loss would explain the frequently observed body weight regain. It should be noted, however, that in other cases only a temporary drop in RMR has been observed [64]. Furthermore, it has been argued that the decreased metabolic rate after weight loss is not different from metabolic rate of lean individuals with the same body weight [64,65] or that it prevented weight stability [66]. Furthermore, a lower RMR did not predict body weight re-gain [64]. In summary, it is clear that weight loss is accompanied by a decrease in metabolic rate, but it is unclear how long this persists and to what extent this contributes to weight regain. It is clear that feedback mechanisms operate, but it is not clear to what extent these are acute or long term and nor is it clear whether these respond to body weight as a cue.

The disproportional reduction in metabolic rate in reaction to energy restriction is accompanied by a number of metabolic alterations. In particular, leptin levels have been shown to drop more than would be predicted by the reduction in body weight [14,67]. It has been suggested that this disproportional reduction of leptin would lead to a deficiency of leptin in the brain, which creates a risk for increased appetite in the weight-reduced state [68]. Leptin administration can counteract adaptations of metabolism to body weight loss [68], but also in this case it is unclear whether this affects long term body weight regain. When we look at body weight loss and leptin levels in mice undergoing body weight loss by different diets, either a low fat diet or restriction of a high fat diet, we observed that epididymal white adipose tissue mass was reduced by 55% and 73%, respectively, while leptin levels were reduced by 73% and 76%, respectively [49]. This may indicate that the diet macronutrient composition can alter the relative reduction in leptin levels. These results amplify the need to understand the extent and duration of disproportional reductions of metabolic rate and/or leptin levels. The same is true for other mechanisms that were shown to accompany body weight loss and may predispose to body weight regain, such as altered fat oxidation and adipocyte hyperplasia [69], as well as increased appetite [54,70] and altered food preference [71]. However, it should be realized that if food/energy intake will match metabolic rate/energy expenditure, it will not predispose to body weight regain [64]. A balanced diet is therefore of utmost importance.

## 11. Metabolic Flexibility

Many different body weight loss strategies are advocated, but most have not been thoroughly tested. An example is the effect on body weight loss that has been attributed to having a breakfast (or not). The effect of breakfasting on body weight was tested only recently and no discernable effect on body weight was observed [72]. This study examined an effect on body weight, but did not examine whether there was an effect of “periodic eating” on health. That periodism of eating may affect health parameters is suggested by beneficial effects that are associated with intermittent fasting [73]. However, studies in women that compared intermittent and continuous weight loss regimens did not only observe an absence in differences in weight loss, but also did also not show a clear reduction in disease risk parameters [74,75]. In part this may be due to absence of distinct markers of disease risk at the start of an intervention. While absence of disease risk markers may be used to define absence of the particular disease, it is less clear how to define health. Recently, metabolic flexibility was proposed as a measure for metabolic health [76,77]. One important characteristic of metabolic flexibility is the ability to switch between different energy sources that are available. How well an individual is able to switch between different nutrient sources can thus be used to characterizes their metabolic health [76]. Substrate switch efficiency has successfully been used to assess the beneficial health effects of dietary interventions [78]. In humans, caloric restriction [79] as well as exercise training [80] seem to increase metabolic flexibility, however, it is currently not known whether different body weight loss strategies exert different effects on substrate switch flexibility, hence, metabolic health.

## 12. Hedonistic Thresholds

The food intake pattern of an individual is a form of behavior, the structure of which can be recognized in frequency of eating and size of portions. This behavioral pattern, together with nutrient content and energy density of the diet, determines the amount of energy that is ingested [81]. Therefore, it seems that we have control of what we eat. However, humans have a hedonistic mind-set, inclined to pursue happiness. Foods rich in sugars and fats offer potent rewards [82], which promote eating even without energetic requirements for food [83]. To some extent, this is true for C57BL/6J mice as well, which gain body weight and develop obesity on a high fat diet [83]. On the other hand, several strains of rats remain as lean on a standard chow diet as when they are fed a pelleted high fat chow diet (e.g., [84]). However, rat strains that remain lean on a chow diet develop obesity when given a cafeteria diet, a diet in which a free choice of different food items, which differ in texture and macronutrient composition, is being offered (e.g., [85,86]). Thus, in mice and rats the pleasure achieved from eating, like in humans, is an important component in body weight gain [87], although a different hedonistic threshold, between species and individuals, may exist.

**Figure 4:**
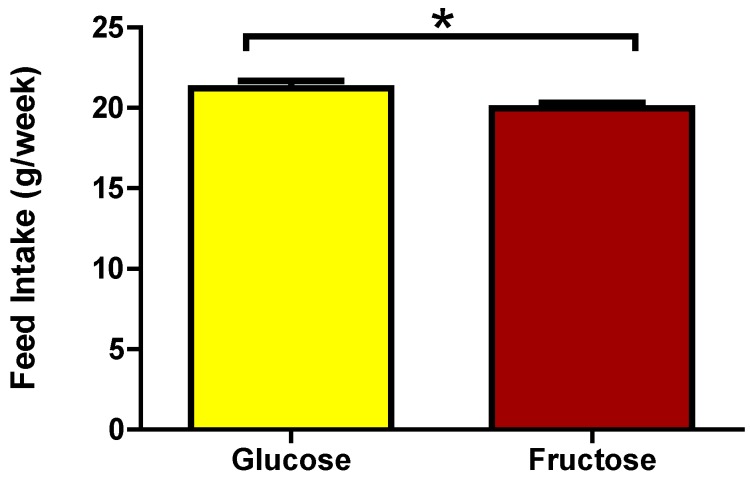
Food intake of C57BL/6J mice on diets with different sugars. C57BL/6JOlaHsd (Harlan) mice (*n* = 12 per group) were fed a purified 36 energy per cent (en%) high fat diet (based on [51]) with 65% (w/w) of carbohydrates present either as glucose or as fructose for six weeks. Mice receive *ad libitum* food and drink and were maintained at 21 °C, 12 h light/12 h dark. Weekly food intake was determined. Data are shown as mean ± SEM. T-statistics (Graphpad Prism) revealed a significant difference (*p* < 0.05) between the group consuming the glucose diet and the group consuming the fructose diet [90].

It is important to realize that differences also exist between energy intake via food or drink. When sweetened drinking water, rather than water alone, is supplied in addition to water and *ad libitum* food, rats and mice consume more energy and display increased metabolic complications (e.g., [88]). Apparently, energy is differently sensed when provided in a solid form than in a liquid form. It should be noted that a high fat diet is more effective in inducing metabolic complication in C57BL/6J mice, while addition of a high sucrose drink is more effective in rats [88], in agreement with differences in the hedonistic threshold described above. In drink, glucose is also differently sensed compared to fructose or a mixture of glucose and fructose, with less of the glucose drink being consumed [89]. Our own data may suggest that this is not the case for feed. When C57BL/6J mice were fed a 36 energy % high fat diet with 65% of carbohydrates being present as glucose (with the remainder as starch) or the same diet in which glucose was replace by fructose, the lowest food intake was observed for the fructose diet (Figure 4).

In summary, different hedonistic thresholds with respect to food choice and food texture exists. This is also the case for humans [91,92]. While in the scientific literature emphasis is on food intake, it should be realized that drink is also an important component of our diet and that large differences exist between the perception of food and drink, for example in terms of satiety [93].

## 13. Nutrigenomics Approaches

Although many studies have examined body weight loss and body weight regain, many aspects remain elusive. In particular, relatively little is known about the molecular changes that accompany body weight loss and body weight regain and how they are related to changes in RMR. Similarly, hardly any controlled mechanistic and physiological studies are available on repeated weight loss and regain. Understanding these processes in molecular detail seems key to the delineation of contributors to the bodyweight settling point. Large scale unbiased techniques are ideally suited for analysing this [67,94]. Transcriptome analysis, employing whole genome gene expression microarrays, can be used to assess changes in expression of protein-encoding mRNAs, long non-coding RNAs (lncRNAs) and micro-RNAs (miRNAs) on a global scale [95,96,97,98,99]. In the past 15 years, DNA microarray technology has developed into a robust technology, with many of the initial bioinformatical hurdles being taken [100]. This makes the technique well suited, in particular, for comparative analyses. At present, large scale RNA sequencing is increasingly being used, with the advantage of broader coverage [101]. Bioinformatical analysis, however, remains a major challenge. Proteomic studies are a major complementary tool and especially relevant to support transcriptome studies at a functional level and to provide insight in the role of secondary protein modifications, which increasingly emerges as an important level of regulation (e.g., [102,103]). The relatively limited coverage, and the demanding efforts to assure reproducibility [104], limit the application of proteome analysis to dedicated research groups with a specific focus. Metabolomics, especially because of major developments in mass spectrometry equipment [105,106], is increasingly being used to complement studies at the mRNA or protein level [107,108]. In particular, the combination of various technologies (e.g., transcriptomics and metabolomics) allows for improved functional interpretation [109,110,111]. Despite the power of ‘omics technologies, it should be realized that they only have their relevance in the context of physiological alterations. Furthermore, when effects have been observed, validation is important using distinct techniques, including targeted biochemical assays and immunohistochemistry, as well as gene-disruption and over expression experiments. Changes that occur during body weight loss and body weight regain in key metabolic organs, such a muscle, adipose tissue, liver and intestine, and the brain, can easily be studied in animal models, but most of these tissues are hardly accessible in humans. Comparative analyses of serum and plasma is key for the translation of results obtained in rodents to humans. Metabolomics analysis allows the identification of metabolite profiles which facilitate substantially improved interpretation of changes in metabolism compared to the analysis of individual metabolites [108]. This is complemented by analysis of changes in the plasma and serum proteome. Recently, for example, we have observed that changes in oxidative stress gene expression profile in WAT under different dietary conditions, is reflected by a serum marker for protein oxidation [112]. As an alternative to metabolome and proteome analysis of serum/plasma, transcriptome analysis of peripheral blood mononuclear cells (PBMC) is increasingly used [113,114]. This can be complemented by PBMC proteome analysis [104,115]. PBMC can be obtained both in humans and rodents and are ideally suited for comparative analysis, especially because gene expression changes that occur in PBMC were shown to reflect changes that occur in target tissues, such as the liver [85]*.*

## 14. The Next Steps

**Figure 5:**
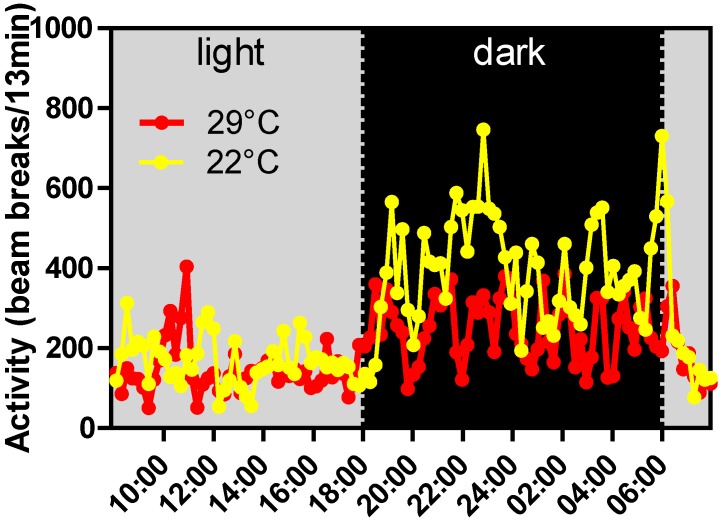
Mean activity of C57BL/6J mice at 22 °C and 29 °C. C57BL/6JOlaHsd (Harlan) mice kept at 29 °C (thermoneutrality) or 22 °C, with *ad libitum* food and drink and 12h light/12 h dark. Mean activity (*n* = 8 per group) has been determined using infrared XY beam beaks (TSE systems) [120].

Carefully controlled experiments in rodents are essential to be able to delineate the various contributors. In delineating contributors, it should be realized that the conditions under which most rodent experiments are performed, being a fixed environment without food selection choice is clearly not reflecting humans under free living conditions. That this may have important metabolic consequences is exemplified by a study in which periodic high dietary cholesterol intake was shown to be less damaging than continuous dietary intake of the same total amount of cholesterol [116]. Therefore, we should start to think how we can mimic more natural variation in eating behavior, while maintaining high levels of control. We should not only think about dietary regimens, but we should also carefully think about the environmental conditions that are being used, including housing temperature. It is currently debated which ambient temperature is best to compare mouse to human (e.g., [117] *versus* [118]). This is relevant because ambient temperature affects many aspects of metabolism and behavior. For example, the effect on body weight of uncoupling protein 1 ablated mice only became apparent under thermoneutral conditions [119]. While thermoneutrality decreases metabolic rate, due to decreased non-shivering thermogenesis, it also decreases activity in the dark (active) period (Figure 5). Also, under controlled conditions it is important to facilitate natural conduct as much as possible, for example by providing sufficient bedding to accommodate differences in day time and night time behavior.

Finally, animal studies may help to better understand how diet interacts with genotype. For example, while C57BL/6J mice are susceptible to diet induced obesity, A/J mice are resistant. Obesity resistance in A/J mice is in part due to high fat diet-induced non-shivering thermogenesis and muscle lipid oxidation [121], showing that differences between strains can be used to better understand diet-genotype interactions. Studies using animal models also allow the use of genetic modification to examine the role of individual genes in body weight regulation in a tissue and temporal manner. Despite the essential contribution that animal model studies can make, it should always be our goal to delineate contributors to weight gain that are relevant in humans.

## 15. Conclusions

There has been little success in defining a body weight setpoint. Rather, it is more likely that body weight is determined by a settling point, being resultant of many different contributors, probably of different strength and nature. These contributors to a certain degree are also likely to differ for each individual. Successful body weight loss strategies will depend on identifying the main contributors and examining to what extent they are important for body weight maintenance in a certain individual.

Established contributors to body weight regulation may be used as functional targets for nutrients. Nutrigenomic technologies are powerful tools both for identification of contributors to body weight regulation as for functional screening of potential beneficial nutrients.
